# Association of the Depth of Invasion With Recurrence Rates of Basal Cell Carcinoma in a Tertiary Health Care Facility: A Retrospective Study Over a Period of Six Years

**DOI:** 10.7759/cureus.36276

**Published:** 2023-03-17

**Authors:** Ambreen Unar, Hira Khan, Nida Zahid, Mustafa A Khan, Saira Fatima, Safdar A Shaikh, Mohammad Fazlur Rahman

**Affiliations:** 1 Otolaryngology - Head and Neck Surgery, Aga Khan Health Service Pakistan, Karachi, PAK; 2 Otolaryngology - Head and Neck Surgery, Aga Khan University Medical College, Karachi, PAK; 3 Epidemiology and Public Health, Aga Khan University Hospital, Karachi, PAK; 4 Medicine, Aga Khan University Hospital, Karachi, PAK; 5 Histopathology, Aga Khan University Hospital, Karachi, PAK; 6 Surgery, Aga Khan University Hospital, Karachi, PAK; 7 Plastic Surgery, Aga Khan University Hospital, Karachi, PAK

**Keywords:** clinical audit, tertiary care center, surgical skin excision, recurrence rate, basal cell carcinoma histopathology

## Abstract

Background

Basal cell carcinoma (BCC) is one of the most common types of cutaneous malignancies and the most frequently occurring form of cancer worldwide. The incidence of basal cell carcinoma is difficult to determine due to its wide geographic variations; however, it has been increasing worldwide with an annual increase of 7% in the number of reported cases. Although BCC is more prevalent in the aging population, diagnosis in younger individuals is steadily increasing. BCC has overall low mortality, however, it leads to significant economic and physical impact on patients and their families along with adding burden to the healthcare system. The primary risk factor for the development of BCC is increased cumulative sun exposure, particularly to UV radiation. The UV index of Karachi averages around 12 (extremely high) during summer months, putting the population at a significantly higher risk of developing BCC in the long term.

Objectives

This audit was undertaken with the following primary objectives: to use the data collected to determine possible prognostic factors for BCC, to measure the rate of recurrence and the number of new primary tumors detected, to study the completeness of follow-up by patients, and to co-relate histopathological findings with the recurrence rate of basal cell carcinoma.

Methods

A retrospective analysis was performed for all patients with BCC who had undergone surgical resection over a six-year time period. Patient charts were reviewed for demographic information, tumor size, onset-to-diagnosis, anatomic location, clinical subtype, histologic differentiation, method of surgical treatment, and recurrence. Data were entered and analyzed in SPSS version 23 (IBM Corp., Armonk, NY).

Results

The review identified cases of BCC in 99 patients. Of the 99 patients, 60.39% were men and 38.38% were women. The most frequent age group was 65-85-year-olds (42 patients, 42.85%) for BCC. Based on the aesthetic units of the face, the most common location was the nasal unit (30 cases, 30.30%) for BCC. Most of the lesions were closed primarily; however; local flaps were used in the case of surgical defects. The recurrence rate was 19.19% for BCC in this study. Our study included 1.0% of patients who were classified as Clark classification level 2 of BCC, 6.1% as Clark level 3, 23.4% as Clark level 4, and 0.16% as Clark level 5. Recurrence rates were seen to increase with increasing Clark classification level in this study.

Conclusion

In our study, many characteristics of BCC were compared to previously published reports and the results were seen to be generally similar. This study correlates the recurrence of BCC with Clark’s classification, showing that depth of invasion is a significant factor in predicting recurrence. There is a paucity of literature regarding the depth of invasion of BCC along with its’ Clarks classification and recurrence. Further studies can help explore and establish the characteristics of BCC.

## Introduction

Basal cell carcinoma (BCC) is a slow-growing epidermal malignancy arising from the basal layer of the epidermis and its appendages. Metastasis is extremely rare, but the disease can spread to regional lymph nodes, lungs, and bones [1]. BCC is the most common type of cutaneous malignancy seen around the world. Its incidence is increasing worldwide, with the literature reporting an annual increase of BCC of 7% for both genders [2]. In 2002, the incidence was 884 in 100,000, which, in 2011, increased to 1840 in 100,000 [3]. Out of all countries, Australia has the highest rate of basal cell carcinoma among its population [1]. The five-year reoccurrence rate of basal cell carcinoma after surgical intervention is 3.2% to 8% [4].

Morbidity associated with basal cell carcinoma results from local tissue invasion and destruction, particularly in the head and neck. Perivascular and perineural invasion are features associated with highly aggressive tumors. Mortality is becoming exceedingly rare, and the median age of death is higher than that of squamous cell carcinoma. Risk factors associated with basal cell carcinoma are excessive exposure to ultraviolet radiation, previous non-ionizing radiation, human papillomavirus (HPV) infection, mutations in genes such as p53 and PTCH, skin types I and II, immunosuppression, and arsenic exposure [5]. Anatomically, 93% of primary basal cell carcinomas occur in the head and neck region with the nose being the most common site while the other 7% arise in the trunk. There are different morphological and histological variants of basal cell carcinoma: nodular, superficial, sclerosing, pigmented, trabecular, and adnexal, of which the most common subtype is nodular [5].

Common treatment modalities for basal cell carcinoma are surgical excision and minimally invasive destructive procedures. Radiotherapy can be considered when surgery is not feasible for locally advanced tumors. Targeted chemotherapy, such as Sonic Hedgehog pathway inhibitors, is used for metastatic or locally advanced basal cell carcinoma that cannot be treated with surgery or radiation [6,7]. Locally aggressive basal cell carcinoma of head and neck patients experience a high rate of local recurrence but low disease-specific mortality when treated with primary surgery and selected use of adjuvant treatment [8,9].

The aims of our study were to conduct an audit and determine the prognostic factors for BCCs along with their frequencies, to measure the rate of recurrence and the number of primary tumors detected, and to determine the completeness of follow-up by patients. We also aimed to discover and correlate any risk factors with the recurrence of disease in our patients.

## Materials and methods

A retrospective consecutive case-series study, including all patients with biopsy-proven BCC from 2014-2019, was conducted after Ethics Review Committee (ERC) consent from our institution. We reviewed 99 patients who received treatment for BCC at the department of surgery in Aga Khan University Hospital (AKUH), Karachi. Patients between the ages of 18 and 60 years were included in the study. However, patients with incomplete information, patients with other cutaneous malignancies, and patients with radiation-induced basal cell carcinoma were excluded from this study. Data were collected after ethical review committee exemption. Information of all the patients who came to AKUH with basal cell carcinoma and met our eligibility criteria was recorded by the research team from their medical records on a predesigned proforma. Data were collected on documentation of pre and postoperative risk factors, treatments received, and subsequent follow-up. Patient records were reviewed for their demographic information, dimensions of the tumor, anatomical location of the tumor, clinical classification and subtype, histologic features, choice of method for surgical treatment, and tumor recurrence. Information was collected by the research team on the following: patient’s age, gender, and reason for the hospital visit, treatment modality received by the patient’s excision with or without radiation, site of the tumor, size of the tumor and width of the margins, and recurrence of the disease at the two-year follow-up. Ethical exemption was taken from the institutional ethical review board. Data were entered and analyzed in Statistical Program for Social Sciences (SPSS ) version 23 (IBM Corp., Armonk, NY). Descriptive statistics were reported as frequency and percentages for categorical variables, which were analyzed using the chi-square/Fisher exact test. Quantitative variables were reported as appropriate and are assessed by the independent t-test/Mann-Whitney U test. A p-value of <0.05 was considered significant.

## Results

**Figure 1:**
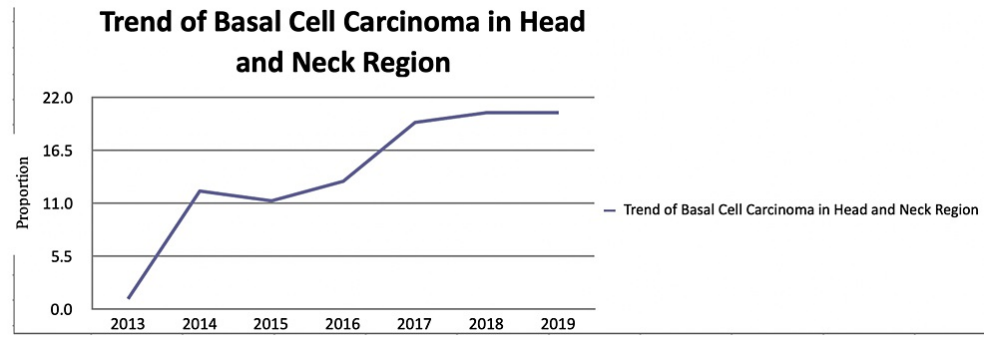
Number of patients who received treatment for basal cell carcinoma at our institute from 2014-2019

**Figure 2:**
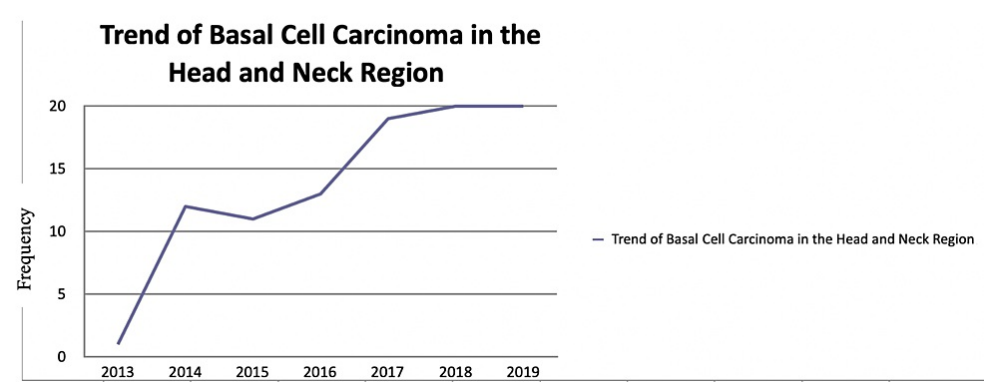
The overall rate of incomplete excisions was 4.6%

We retrospectively reviewed the charts of the 99 patients from 2014 to 2020 who received treatment for basal cell carcinoma at our institute. Out of these, 74 were excised by plastic surgeons, five were resected by ENT surgeons, and the remaining were removed by other specialties. In our study, 60.39% of patients were men and 38.38% were women. Patient ages ranged from 29 years to 89 years, with a mean ± SD age of 63.02 ± 14.99 years. The number of patients managed and treated along the lines of BCC at our institution increased each year during the study period as shown in Figures 1-2.

Tumor size

 The median tumor diameter was 3.92 cm with an interquartile range of 2.03-8.45 as shown in Table 1. The median anteroposterior diameter of tumors was 2.00 mm and the median transverse diameter was 2.45 mm.

**Table 1:** Showing tumor size, depth of invasion, and diameter of the tumor

		Tumour Size (mm^2)	Depth	Transverse Dimension	Vertical Dimension
N	Valid	88	58	88	88
	Missing	11	40	10	10
Mean		9.2289	.433	2.8284	2.2295
Median		3.9200	.300	2.4500	2.0000
Std. Deviation		16.19671	.4050	1.91058	1.72054
Percentiles	25	2.0250	.200	1.6000	1.0250
	50	3.9200	.300	2.4500	2.0000
	75	8.4450	.500	3.5000	2.5000

Anatomic distribution

Preoperative photographs were taken into consideration and reviewed to analyze the location of the tumors and were subsequently categorized based on different aesthetic units of the face (Table 2). The results showed a predilection for BCC for the nasal unit (30 cases, 30.30%), followed by the cheek (20 cases, 20.20%) and the forehead (15 cases, 15.15%).

**Table 2:** Distribution of basal cell carcinoma by aesthetic units of the head and neck region

		Frequency	Percent
	Total	99	100
Nose	Yes	30	30.30
Nasolabial fold	Yes	6	6.06
Lateral canthus of eye	Yes	13	13.13
Medial Canthus of the eye	Yes	5	5.05
Cheek	Yes	20	20.20
Periauricular region	Yes	6	6.06
Lip	Yes	4	4.04
Forehead	Yes	15	15.15

Surgical treatment: operative methods

Adhering to our inclusion criteria, all the patients were treated with wide local excision of the tumors as the first choice of management. The most common method was primary closure of the lesion (68.0%), followed by a local flap (21.4%), and the rest of the cases included closure by other methods.

Recurrence

Of the 99 cases, local recurrence was noted to have occurred in 19 cases (19.19%) during the six-year study period. Patients who underwent a secondary operation were reported to have a curative rate without recurrence of 22.44%. However, there was one patient who experienced a recurrence of BCC after the secondary operation but was lost to follow-up (Table 3).

**Table 3:** Analysis of the relationship of the margin status with recurrence

			Recurrence			Total	
			N/A	Yes	No		
Surgical Margins	N/A	Count	0	1	2	3	
		% within Recurrence	0.0%	5.3%	2.7%	3.0%	
	>5 mm	Count	1	7	35	43	
		% within Recurrence	16.7%	36.8%	47.3%	43.4%	
	< 5 mm	Count	5	11	37	53	
		% within Recurrence	83.3%	57.9%	50.0%	53.5%	
Total		Count	6	19	74	99	

Clark's classification in relation to the recurrence of BCC in patients

We evaluated the histopathological variables in a total of 46 patients, 39 of which experienced a recurrence of BCC. Fifty percent of the cases were found to have Grade IV Clark's classification and and 34.8% were in the Grade V category (Table 4). The percentage of recurrence was seen to increase in patients who had a higher Clark's classification of lesions.

**Table 4:** Distribution of basal cell carcinoma according to Clark's classification and the percentage of recurrences in 50% of cases

Clark's classification	Number of patients	% Recurrence
2	1	0
3	6	0
4	23	8.7
5	16	25

## Discussion

We conducted this study in order to audit the patients with BCC in our setting and compare outcomes and prognostic factors with those seen in the literature. Basal cell carcinoma is the most common cutaneous malignancy around the world with incidence increasing worldwide over the last decade [10]. It is more prevalent in the elderly population, which is consistent with our study [11]. A number of studies conducted in Korea have reported increases in the overall incidence of cutaneous malignancies, with the study by Kim et al. demonstrating an increase from 0.25% to 0.34% over a period of just 10 years [12]. In previous studies, the incidence of cutaneous malignancies has been shown to be greater for males than females [13], but recent studies have shown otherwise [14].

The ideal surgical treatment for BCC is complete removal, and it can be achieved with safe excision margins [15]. A 4-mm surgical margin of clinically normal skin is required for an elliptical excision, which is the most often used treatment for basal cell carcinoma. However, because of the cosmetic limitations of the face, a 4-mm surgical margin is often not compatible with the goal of holistic patient care [15].

Surgical excision is an effective treatment option for BCCs with reported recurrence rates of less than 5% [16]. Combination curettage and excision was the most often utilized treatment method for patients. A low recurrence rate of 10.5% has been observed following aggressive curettage and excision [17].

In the present study, the median tumor diameter was 0.25 cm for BCC. The face is more prone to develop skin cancer because it receives the most cumulative amount of UV radiation throughout the day. In fact, most BCC cases are found in the head and neck region [18], which is consistent with the results of our study.

Patients having a history of several BCCs are more likely to experience further skin malignancies in the future [19]. In this audit, more than one-third of individuals who had new lesions also had one or more prior BCCs. Longer follow-up was typically required for lesions that were incompletely excised, aggressive in histology, or located in high-risk locations. All of these have a poor prognosis and an increased risk of recurrence. This audit looked at patients who had their primary BCC treated by simple surgical excision and found that there was inadequate documentation of lesion size and the histological features of the tumors (only aggressive lesions were documented).

The majority of plastic surgeons acknowledge that patient follow-up has recognizable benefits, however, the desire for routine follow-up was not expressed by 62% of patients. Our clinic's high percentage of follow-up appointment cancellations leads us to believe that some of our patients would prefer not to receive regular follow-ups. Loss of compliance is caused by the costs associated with patients skipping review appointments. The cost of follow-up attendance at outpatients is difficult to estimate but by applying current figures to our audit, the cost of two years of follow-up per patient is Rs. 12000 (USD 150), while the cost of one year of follow-up is Rs. 6000 (USD 75). This proves to be substantial, especially in Pakistan where the most common means of paying for healthcare is out-of-pocket.

We reviewed the literature and found some regional and international work with similar ideas as shown in Table 5. The rate of recurrence in our retrospective review of the chart was 19.38%, which is consistent with studies conducted in Sweden, Turkey, the Netherlands, and the United Kingdom [8,20-22]. According to our audit, the nodular type is the most commonly prevalent type of basal cell carcinoma in our population, which is consistent with the results of audits conducted in other studies except Elis Salimi et al. [23], who concluded that the infiltrating type is the most common in their region. The most common region involved in basal cell carcinoma is the head and neck, which is in concurrence with our findings.

**Table 5:** The literature review of international audits on basal cell carcinoma and comparison of results with our study

Author and year of publication	Number of patients	Duration of follow-up	Rate of recurrence	Country	Recommended resection margins	Most common location ( head and neck )	Mean age of patients	Most common histological subtype
Mc Loone et al. 2006 [22]	114	1999-2000	Low Risk of developing new 11-63%	Belfast, United Kingdom	2 cm	Yes	70 years	Nodular
Venables ZC et al. 2019 [24]	285	2013-2015	No comment	Wales, United Kingdom	2 cm	Yes	71 years	Nodular
Kyung Won Kang et al. [12]	265	19 years	3.9%	Korea	-	Yes	70-79 years	Nodular
Elias Salimi 2017 [23]	59	-	1.9%	Iran	2 mm - 4 mm	Yes	69.9 years	Infiltrative type
Eva Van Loo [21]	469	1999-2002	13.3%-56% (5-year follow-up)	The Netherlands	5 mm - 2 cm	Yes	No comments by the author	Nodular
J. Kappelin 2020 [8]	3911	2008-2015	5%	Sweden	2 cm	Yes	72 years	Nodular
Sibel Hakarverdi et al. 2011 [2]	197	2005-2010	17.7%	Turkey	2 cm	Yes	64.11 years	Nodular
AKUH 2021 (current study)	98	2014-2019	19%	Karachi, Pakistan	5 mm - 2 cm	Yes	63 years	Nodular

BCC is associated with genetic syndromes such as Bazex syndrome, basal cell nevus syndrome (also called Gorlin syndrome), and xeroderma pigmentosum [25]. Our study found three patients with co-existing conditions with BCC including melanoma, osteosclerosis, and myelofibrosis.

Many factors are associated with the recurrence of basal cell carcinoma. These include aggressive clinical and histopathologic features like morpheiform, perineural invasion, irregular border), immunosuppressive status, or prior field irradiation [12]. Lesion size is also a factor (>10 mm on a cheek, forehead, scalp, or neck, or of >6 mm on the central face, periorbital area, nose, lip, chin, or auricle). Out of 19 cases of recurrence in our study, 13 tumors were resected by plastic surgeons.

In our study, perineural involvement was not present in any tumors. However, lymphovascular invasion was present in two patients.

Strengths and limitations

The present study evaluates patients of BCC at the department of surgery from 2014 to 2019. Our registry has a near-complete coverage of performed surgeries, thus, the registry has a representative coverage of BCC tumors treated in the area during this period of time, hence decreasing the risk of selection bias. A majority of excisions were made by plastic surgeons and followed up by otorhinolaryngologists. Furthermore, this study presents detailed data regarding surgical margins in relation to histological subtypes and tumor sites. The strengths of our study include the sample size, which is that of international standards, and this is the first audit to investigate BCC at our institute. The limitations include being a single-center study design, which leads to non-generalizable results in the Pakistani population. Our study was retrospective, therefore, we encountered some missing data, which had to be excluded.

## Conclusions

Basal cell carcinoma continues to be the most frequently occurring cutaneous malignancy worldwide. The data collected in this study will serve as a reference for future research and the development of preventive strategies in our population. This study also highlights the lack of proper documentation of lesion description and the loss to follow-up seen in patients treated for BCC. Follow-up proves to be vital in healthcare, as it allows physicians to re-evaluate patients following treatment and detect recurrences earlier.
